# Vitamin D deficiency is an independent predictor of mortality in patients with chronic heart failure

**DOI:** 10.1007/s00394-018-1806-y

**Published:** 2018-08-18

**Authors:** Richard M. Cubbon, Judith E. Lowry, Michael Drozd, Marlous Hall, John Gierula, Maria F. Paton, Rowena Byrom, Lorraine C. Kearney, Julian H. Barth, Mark T. Kearney, Klaus K. Witte

**Affiliations:** 1 Division of Cardiovascular and Diabetes Research, Multidisciplinary Cardiovascular Research Centre (MCRC), Leeds Institute of Cardiovascular and Metabolic Medicine, University of Leeds, LIGHT building, Clarendon Way, Leeds, LS2 9JT UK; 2 Department of Clinical Biochemistry, Leeds Teaching Hospitals NHS Trust, Leeds, UK

**Keywords:** Vitamin D, Chronic heart failure, Mortality

## Abstract

**Purpose:**

Low 25-hydroxyvitamin D (25[OH]D) concentrations have been associated with adverse outcomes in selected populations with established chronic heart failure (CHF). However, it remains unclear whether 25[OH]D deficiency is associated with mortality and hospitalisation in unselected patients receiving contemporary medical and device therapy for CHF.

**Methods:**

We prospectively examined the prevalence and correlates of 25[OH]D deficiency in 1802 ambulatory patients with CHF due to left ventricular systolic dysfunction (left ventricular ejection fraction ≤ 45%) attending heart failure clinics in the north of England.

**Results:**

73% of patients were deficient in 25[OH]D (< 50 nmol/L). 25[OH]D deficiency was associated with male sex, diabetes, lower serum sodium, higher heart rate, and greater diuretic requirement. During a mean follow-up period of 4 years, each 2.72-fold increment in 25[OH]D concentration (for example from 32 to 87 nmol/L) is associated with 14% lower all-cause mortality (95% confidence interval (CI) 1, 26%; *p* = 0.04), after accounting for potential confounding factors.

**Conclusions:**

Low 25-hydroxyvitamin D deficiency is associated with increased mortality in patients with chronic heart failure due to left ventricular systolic dysfunction. Whether vitamin D supplementation will improve outcomes is, as yet, unproven.

**Electronic supplementary material:**

The online version of this article (10.1007/s00394-018-1806-y) contains supplementary material, which is available to authorized users.

## Introduction

Vitamin D is receiving increasing attention as epidemiological evidence links it to many chronic illnesses. Low serum 25-hydroxyvitamin D (25[OH]D) concentrations have been associated with an increased risk of adverse outcomes generally, [1, 2] but especially in patients with suspected coronary artery disease, hypertension and chronic heart failure (CHF) [3–10]. However, published data pertaining to CHF are derived from registry data, or somewhat selected cohorts such as patients referred for coronary angiography, on a waiting list for cardiac transplantation or in-patients. Vitamin D has multiple effects throughout the body that may be of particular relevance in people with CHF [11]. We have recently described in a randomised, placebo-controlled trial in unselected ambulatory patients with CHF and 25[OH]D deficiency that vitamin D_3_ supplementation (100 mcg (4000 IU) daily) improves left ventricular structure and function [12]. Whether 25[OH]D deficiency independently predicts outcomes in unselected patients with CHF remains unclear.

In a prospective cohort study, we examined the prevalence and correlates of 25[OH]D deficiency and whether 25[OH]D levels were related to hospitalisation and mortality outcomes in unselected patients referred to heart failure clinics in the north of England.

## Methods

As described in our previous publications [13, 14], adult patients (> 18 years) were eligible to take part in the cohort study if they had stable symptoms and signs of CHF for 3 months, in addition to echocardiographic evidence of left ventricular systolic dysfunction (left ventricular (LV) ejection fraction (LVEF) ≤ 45%). Between June 2006 and July 2014, we recruited patients from the outpatient heart failure clinics of four hospitals in the north of England. Ethical approval was provided by Leeds West Research Ethics Committee (07/Q1205/17), and all patients provided written informed consent to participate.

### Baseline assessment

All patients underwent resting 12-lead electrocardiographs, and blood testing for measurement of full blood count, electrolytes, serum creatinine and serum 25(OH)D_2_ and 25(OH)D_3_ concentration. Estimated glomerular filtration rate (eGFR) was calculated using the Modification of Diet in Renal Disease method [15]. Functional status was assessed using the New York Heart Association (NYHA) classification. Two-dimensional transthoracic echocardiography was performed in all participants by sonographers blinded to patient characteristics; left ventricular (LV) ejection fraction (LVEF) was calculated according to the Simpson’s biplane method. Doses of diuretic therapy, angiotensin-converting enzyme inhibitors (ACEi), angiotensin receptor blockers (ARB), and beta-blockers were normalised to maximum licensed CHF dose as previously described [10].

### 25[OH]D concentration

Serum 25(OH)D_2_ and 25(OH)D_3_ concentrations were analysed by tandem mass spectrometry. Samples were prepared using a protein precipitation reagent containing deuterated 25(OH)D_3_. The supernatant was analysed on an API5000 LC-MS/MS (AB SCIEX, Warrington, UK) in APCI mode. The inter-assay CV was < 10% at all concentrations ranging from 12 to 159 nmol/L. 25(OH)D_2_ and D_3_ concentrations were summed and reported as 25(OH)D. We defined deficiency of 25[OH]D concentrations based upon the threshold outlined by the Endocrine Society calling 25[OH]D < 50 nmol/L deficient [16].

### Hospitalisation and mortality

The nature and duration of non-elective hospitalisation within the first year after enrolment was determined using hospital databases. Each hospitalisation was sub-classified independently by two cardiologists as cardiovascular if the principal presenting complaint was related to cardiac, cerebrovascular or peripheral vascular disease; consensus was sought in all initial cases of disagreement. Cardiovascular admissions were further sub-classified as heart failure related if the patient presented with symptoms and signs of heart failure and evidence of fluid overload requiring intravenous diuretic therapy for at least 24 h. All patients were registered with the United Kingdom Office of National Statistics (ONS) to provide details of death until the censoring date of 8th May 2016.

### Statistical methods

Analyses were conducted in Stata (StataCorp. 2015. Stata Statistical Software: Release 14. College Station, TX). All significance tests were two sided and called significant at the 5% level. Continuous data are expressed as mean (SEM) or median (IQR) depending on normality of distribution, and categorical variables as *n* (%).

Odds ratios (binary logistic regression) and hazard ratios (Cox proportional hazards regression) were derived for the association between 25[OH]D and hospitalisation or mortality, respectively. 25[OH]D concentrations were natural log transformed during these analyses to account for their non-normal distribution. After defining crude (unadjusted) associations, models were used to adjust for patient and clinical demographics (age, sex, month and year of recruitment, sodium, eGFR, albumin, log transformed QRS interval, NYHA class, LVEF, LV end diastolic dimension), comorbidities and aetiology (diabetes mellitus, chronic obstructive pulmonary disease (COPD), ischaemic aetiology) and treatment (ramipril dose, bisoprolol dose, furosemide dose and device therapy).

Multiple imputation by chained equations, under the missing at random assumption, was used to impute 30 sets of data to minimise any potential bias caused by missing data (see Table 1) [17, 18]. The imputation model specification was such that it included all variables included in the analysis model, including outcome variables (hospitalisation, Nelson–Aalen survival estimator and censoring indicator [19]), as well as auxiliary variables as indicated in supplementary table 1, following previously defined methods [20]. Results presented in the manuscript contain estimates averaged over 30 imputed data sets, according to Rubin’s rules, and were compared to complete case analyses (i.e. without imputation), presented in supplementary tables 2 and 3, to check for consistency.

**Table 1:** Variables according to 25[OH]D concentration (≥/<50 nmol/l)

	Whole cohort	25[OH]D < 50	25[OH]D ≥ 50	*p* value	Missing
	*n* = 1802	*n* = 914	*n* = 338	25[OH]D groups	*n* (%)
Age (years)	69.6 (12.5)	69.9 (12.7)	71.2 (11.2)	0.07	0 (0)
Heart rate (bpm)	75.3 (17.9)	76.4 (18.3)	73.6 (17.2)	0.017	158 (8.8)
QRS interval (ms)	123.2 (31)	123.8 (31.4)	123.7 (31.4)	0.94	157 (8.7)
Haemoglobin (g/dl)	13.5 (1.8)	13.5 (1.8)	13.4 (1.8)	0.35	20 (1.1)
Sodium (mmol/L)	139.4 (3.4)	139.2 (3.5)	139.9 (3)	0.001	4 (0.2)
eGFR (ml/kg/1.73 m^2^)	57.8 (19.7)	58.5 (19.5)	57.3 (20.7)	0.36	8 (0.4)
Albumin (g/l)	43.1 (11.1)	42.8 (3.7)	43.1 (3.8)	0.29	56 (3.1)
LV end diastolic dimension (mm)	57.2 (8.9)	57 (8.6)	56.7 (8.9)	0.53	71 (3.9)
LV ejection fraction (%)	32 (9.5)	31.6 (9.3)	32.2 (9.4)	0.26	45 (2.5)
Ramipril dose (mg/day)	4.9 (3.5)	4.8 (3.6)	5 (3.5)	0.37	5 (0.3)
Bisoprolol dose (mg/day)	3.9 (3.4)	4 (3.4)	4 (3.3)	0.89	5 (0.3)
Prescribed ACEi/ARB (*n*, %)	1626 (90.4)	818 (89.7)	309 (91.4)	0.36	5 (0.3)
Prescribed beta-blocker (*n*, %)	1523 (84.7)	782 (85.7)	289 (85.5)	0.91	5 (0.3)
Prescribed mineralocorticoid receptor antagonist (*n*, %)	689 (38.2)	367 (40.2)	111 (32.8)	0.017	5 (0.3)
Furosemide (mg/day)	51.2 (1.2)	54.4 (1.6)	44.1 (2.6)	0.001	5 (0.3)
Male sex (*n*, %)	1319 (73.2)	678 (74.2)	230 (68)	0.031	0 (0)
Ischaemic aetiology (*n*, %)	1067 (59.2)	544 (59.5)	187 (55.3)	0.18	0 (0)
Diabetes (*n*, %)	504 (28)	287 (31.4)	77 (22.8)	0.003	0 (0)
COPD (*n*, %)	284 (15.8)	144 (15.8)	52 (15.4)	0.87	0 (0)
Device therapy (*n*, %)	504 (28)	247 (27)	109 (32.2)	0.07	0 (0)
NYHA class 1 (*n*, %)	333 (18.5)	132 (14.4)	66 (19.5)	0.05	2 (0.1)
2	912 (50.7)	478 (52.3)	179 (53)		
3	534 (29.7)	294 (32.2)	92 (27.2)		
4	21 (1.2)	10 (1.1)	1 (0.3)		

Continuous data all as mean (SD), categorical data are *n*, %

*eGFR* estimated glomerular filtration rate, *LV* left ventricular, *COPD* chronic obstructive pulmonary disease, *NYHA* New York Heart Association Class

## Results

**Fig. 1:**
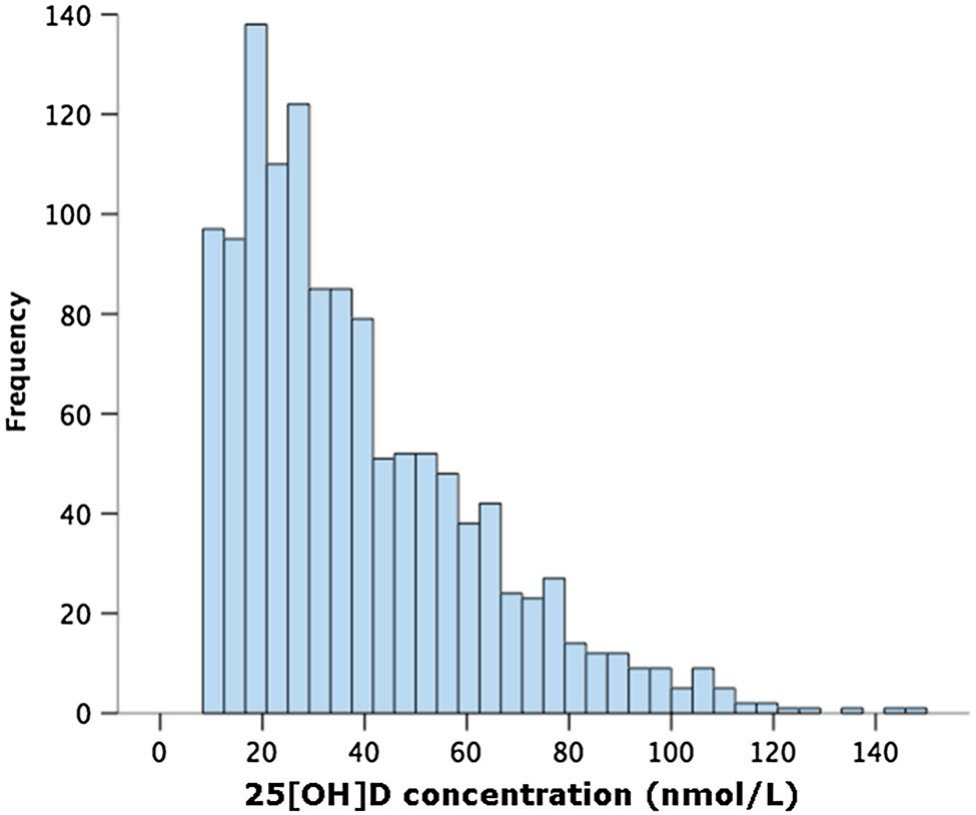
25[OH]D concentrations in patients with chronic heart failure. Distribution of 25(OH)D concentrations in 1252 patients, indicating deficiency (< 50 nmol/L) in 73%

**Fig. 2:**
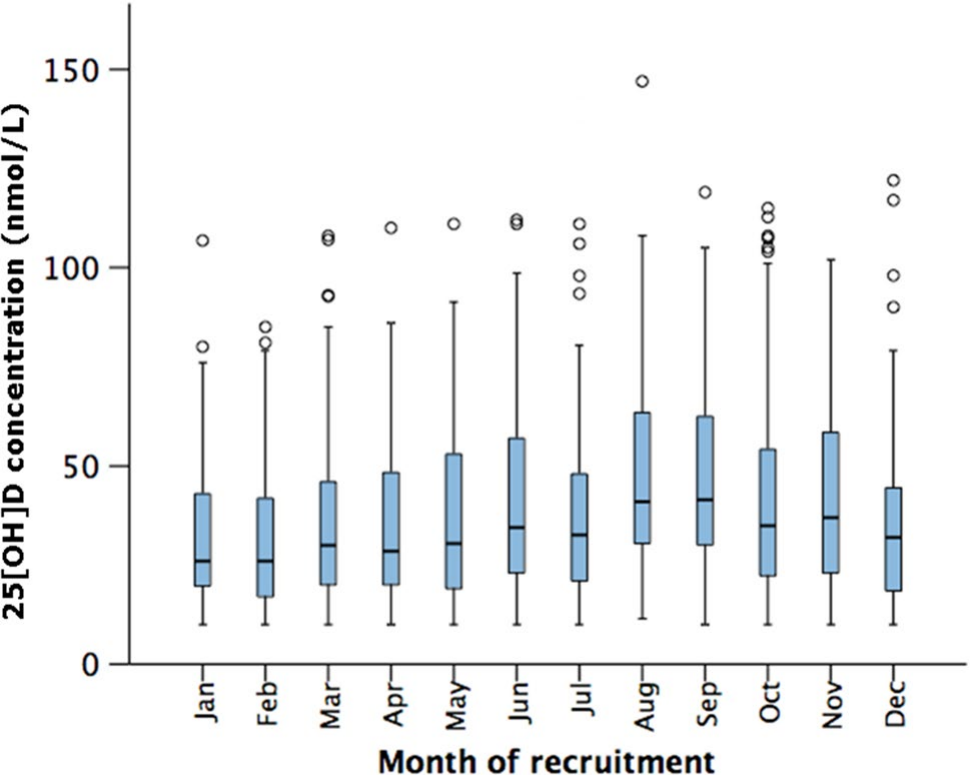
25[OH]D concentrations in patients with chronic heart failure. Monthly variation of 25[OH]D concentration (median and interquartile range) in 1252 patients. Boxes represent median and interquartile range (IQR), with whiskers denoting 1.5 × IQR and circles outliers beyond this range

A total of 1802 patients were recruited to the study [mean (SEM) age 69.6 (0.3) and 1319 (73.2%) male]. Median (IQR) 25[OH]D concentration within the 1252 patients with available data was 32.1 (20–52) nmol/L. Few patients (9.1%) were sufficient in 25[OH]D (≥ 75 nmol/L) at baseline (Fig. 1), and 914 (73%) were deficient (< 50 nmol/L). Although 25[OH]D concentrations varied throughout the year (Fig. 2), even during months with greater daylight, median concentrations remained in the deficiency range (April–September − 35.4 (21.9–56) nmol/L, and October–March − 30 (19.9–48) nmol/L; *p* = 0.001 by Mann–Whitney test).

Patients deficient in 25[OH]D were more likely to be male, and have diabetes, whilst the distribution of heart failure aetiology was not different between the groups (Table 1). Deficient patients also had higher heart rates, lower serum sodium levels, and a greater furosemide requirement. Median 25[OH]D concentrations were significantly related to symptoms: 36.4 (22–57), 33.0 (21–52), 28.5 (19.8–48) and 18.4 (10–42)nmol/L for NYHA classes I–IV, respectively, (*p* = 0.0005 by Kruskal–Wallis test).

### Hospitalisation and mortality outcomes

Using routine data collection, we had 100% follow-up. Table 2 describes the association of natural log-transformed 25[OH]D concentration with hospitalisation during the first year of follow-up, both before and after adjustment for potential confounding factors. Although 25[OH]D concentrations were associated with heart failure-specific, cardiovascular and all-cause hospitalisation in unadjusted analyses, there was no association in all cases after accounting for potential confounding factors. The same conclusions were reached when analyses were repeated without multiple imputation (supplementary table 2).

**Table 2:** Association of 25[OH]D with hospitalisation at 1 year (logistic regression analysis) after multiple imputation

Model	OR^**^	Lower 95% CI	Upper 95% CI	*p* value
Heart failure hospitalisation (*n* = 112, 6.2%)				
Unadjusted	0.67	0.46	0.97	0.034
Adjusted for patient and clinical demographics^a^	0.76	0.52	1.12	0.165
Adjusted for patient and clinical demographics, and comorbidities and aetiology^b^	0.79	0.54	1.16	0.223
Adjusted for patient and clinical demographics, comorbidities and aetiology, and treatment^c^	0.80	0.54	1.19	0.270
Cardiovascular hospitalisation (*n* = 227, 12.6%)				
Unadjusted	0.78	0.59	1.01	0.061
Adjusted for patient and clinical demographics^a^	0.84	0.64	1.10	0.202
Adjusted for patient and clinical demographics, and comorbidities and aetiology^b^	0.86	0.65	1.13	0.280
Adjusted for patient and clinical demographics, comorbidities and aetiology, and treatment^c^	0.87	0.66	1.15	0.330
All non-elective hospitalisations (457, 25.4%)				
Unadjusted	0.78	0.64	0.95	0.011
Adjusted for patient and clinical demographics^a^	0.84	0.68	1.03	0.086
Adjusted for patient and clinical demographics, and comorbidities and aetiology^b^	0.85	0.69	1.04	0.111
Adjusted for patient and clinical demographics, comorbidities and aetiology, and treatment^c^	0.86	0.70	1.05	0.139

Multiple imputation by chained equations was performed with 30 imputations and 20 iterations, and all model estimates are averaged over all imputed datasets

^**^OR per 2.72-fold increase in 25[OH]D (due to natural log transformation to achieve normality)

^a^Including age, sex, month and year of recruitment, sodium, eGFR, albumin, log-transformed QRS interval, NYHA class, LV ejection fraction, LV end diastolic dimension

^b^Diabetes, COPD, ischaemic aetiology

^c^Ramipril dose, bisoprolol dose, furosemide dose, and device therapy

**Fig. 3:**
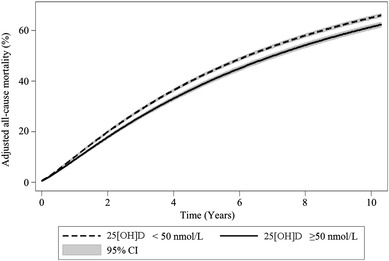
Adjusted survival according to 25[OH]D status. Adjusted survival curves according to 25[OH]D status (deficient and adequate) after multiple imputation, showing that after accounting patient and clinical demographics, comorbidities and aetiology, and treatment factors, 25[OH]D deficiency is associated with decreased survival (HR 1.24, 95% CI 1.05–1.46)

The association between natural log-transformed 25[OH]D and mortality was significant in all of the unadjusted and adjusted analyses (Table 3) with a 19% lower mortality in non-deficient patients. These analyses suggest that even after accounting for all other variables described in Table 1, a 2.72-fold increase in 25[OH]D (for example, an increase of 25[OH]D from 32 to 87 nmol/L) is associated with a 14% lower risk of death. Broadly similar effect size and statistical significance were noted when repeating analyses without multiple imputation (supplementary table 3). Adjusted survival curves with 95% confidence intervals for 25[OH]D deficient and non-deficient patients are shown in Fig. 3, again revealing significantly higher mortality in deficient patients (HR 1.24, 95% CI 1.05, 1.46).

**Table 3:** Association of 25[OH]D with all-cause mortality using Cox proportional hazards modelling after multiple imputation

Model	HR^a^	Lower 95% CI	Upper 95% CI	*p* value
All-cause mortality (*n* = 737, 40.9%)				
Unadjusted	0.79	0.69	0.91	0.001
Adjusted for patient and clinical demographics^b^	0.83	0.72	0.96	0.011
Adjusted for patient and clinical demographics, and comorbidities and aetiology^c^	0.84	0.72	0.97	0.016
Adjusted for patient and clinical demographics, comorbidities and aetiology, and treatment^d^	0.86	0.74	0.99	0.042

Multiple imputation by chained equations was performed with 30 imputations and 20 iterations, and all model estimates are averaged over all imputed datasets

^a^HR per 2.72-fold increase in 25[OH]D (due to natural log transformation to achieve normality)

^b^Including age, sex, month and year of recruitment, sodium, eGFR, albumin, log-transformed QRS interval, NYHA class, LV ejection fraction, LV end diastolic dimension

^c^Diabetes, COPD, Ischaemic aetiology

^d^Ramipril dose, bisoprolol dose, furosemide dose, and device therapy

## Discussion

Our data from a large prospectively collected cohort of unselected ambulatory patients with CHF suggest that 25[OH]D deficiency (25[OH]D < 50 nmol/L) is highly prevalent, persists during summer and autumn, and is an independent predictor of increased mortality. After adjusting for multiple confounders, the outcome data from the cohort study are consistent with previous smaller reports in ambulatory patients [9, 21, 22], hospitalised patients [10] and registry data [3, 6, 23], and reveal increased mortality in patients with low 25[OH]D concentrations compared with those without. In all of these previous studies, 25[OH]D deficiency was less frequent than in our cohort ranging from 28 to 75% [10, 24], whilst overall mortality of the cohorts was higher. Our cohort study was prospectively designed to assess predictors of outcome in an unselected, consecutive group of patients with heart failure due to left ventricular systolic dysfunction on optimal contemporary medical and device therapy, and provides the strongest evidence to date that a low 25[OH]D concentration is an independent predictor of mortality.

### Why might vitamin D be important in chronic heart failure?

Clinical evidence linking micronutrients and cardiovascular disease overall remains neutral [24]. Vitamin D, on the other hand, has multiple effects on the cardiovascular system [25] that might be especially pertinent to the heart failure syndrome [9, 26, 27].

Abnormalities of the vitamin D–parathyroid (PTH) axis have a direct effect upon a wide range of mammalian cells including cardiomyocytes. Through increased urinary excretion of calcium and magnesium, enhanced by loop diuretic use [28, 29], elevated aldosterone levels drive PTH release. This response is exacerbated in people with 25[OH]D deficiency [30, 31]. The consequences of 25[OH]D deficiency [32] and elevated PTH levels [33–36] are calcium loading, with cardiomyocyte and skeletal muscle contractile dysfunction, cellular hypertrophy, oxidative stress, immune activation, endothelial dysfunction (including enhanced endothelin-1 release) [30, 34, 37–41]. These influences are reflected clinically with an increased risk of hospitalisation [7, 42], and worsening renal function [43], whilst vitamin D supplementation may be associated with a reduction of plasma renin and aldosterone levels [44, 45]. Consistent with these observations, we found lower mean sodium levels and higher mean heart rates in 25[OH]D-deficient heart failure patients. Hence, the beneficial remodelling seen in VINDICATE [9], and the adverse effects of 25[OH]D deficiency on outcomes demonstrated in the present report might reflect the interaction of vitamin D with several of the contributory pathophysiological pathways specific to CHF due to left ventricular systolic dysfunction.

### Vitamin D as an intervention

A recent Cochrane review of 56 randomised trials with ~ 95 K participants (> 99% healthy volunteers) showed that vitamin D_3_ (given for mean 4.4 years) decreased all-cause mortality (RR 0.94, 95% CI 0.91–0.98). Vitamin D_2_ had no effect. The authors called for more research in non-healthy populations [46]. A further systematic review and meta-analysis of observational and interventional studies suggested that vitamin D_3_ supplementation might reduce mortality [47], with particular benefit in CHF patients. A subsequent trial analysis, systematic review and meta-analysis suggest that vitamin D supplementation might protect against fatal and non-fatal CHFs in older, healthy people (HR 0.75, 95% CI 0.58–0.97), but not against MI or stroke [48]. The most recent meta-analysis on non-skeletal effects of vitamin D supplementation demonstrates that most trials have been done in subjects without low 25[OH]D levels limiting the credibility of the argument that vitamin D supplementation has no potential benefit on outcomes in people with 25[OH]D insufficiency [1].

The Vitamin D Assessment study (ViDA) in 5100 healthy subjects, aged > 50 years, recently reported that 100,000 IU oral vitamin D_3_ monthly was neutral for the prevention of CV disease [49, 50], with a low rate of 25[OH]D deficiency in participants (25%), a lower than expected endpoint rate and monthly doses cited as possible reasons [51]. Meta-analysis and data from ViDA and other studies describe that benefits on clinical outcomes are greatest in the most deficient, and in studies using daily dosing regimens (as in VINDICATE) rather than monthly [52–55].

Early trials of vitamin D supplementation in CHF were inconclusive possibly due to inclusion criteria, calcium-based placebo, dosing regimen, use of vitamin D_2_ (rather than D_3_) and heterogeneous CHF population [56–58], limiting their ability to provide clarity of benefit [25], whilst no trials have shown any adverse safety signals including up to 10,000 IU daily [59]. Recent meta-analyses have suggested that vitamin D might reduce inflammation (assessed by tumour necrosis factor-alpha levels (TNF-α) [60]), but that it is neutral for heart function [61]. However, the latter analysis did not include data from the VINDICATE paper which presented data from two independent randomised, placebo-controlled, double-blind, parallel group studies using two imaging modalities demonstrating consistent improvements in left ventricular structure and function [9].

The single-centre ‘Effect of Vitamin D on all-cause mortality in heart failure’ (EVITA) study is the only study of patient-orientated outcomes in CHF that has been reported. EVITA was neutral for mortality and heart failure hospitalisation [62]. EVITA recruited only 400 of an intended 950 subjects (which would have given 80% power to detect a 36% reduction in total mortality) and had a drop-out rate of 42% at 3 years. Moreover, the median age of participants was 54yrs, with an unusually high (80%) device therapy rate, the study included people who were sufficient in 25[OH]D (concentrations 50–75 mmol/L) and the group assigned vitamin D supplementation had significantly worse renal function and was 2 years older. In contrast, participants in our two studies and the present cohort study were ambulant outpatients, mean age 70 years with a more typical device therapy rate (30%) and all had 25[OH]D concentrations < 50 nmol/L [12].

## Limitations

Although the results from our cohort study are significant despite the inclusion of numerous potential confounders in the models presented, our cohort outcome data remain observational. We accept, therefore, that it is possible that we have not accounted for all relevant confounding variables including, for example, social background and lifestyle that are difficult to measure. For example, the main source of vitamin D is not nutritional [63], rather the result of skin sunlight exposure. Hence, patients with chronic disease, immobility and the elderly (who require more sun exposure to make the same amount of vitamin D as younger individuals) are at higher risk of 25[OH]D deficiency since they spend less time outdoors [64]. 25[OH]D deficiency could, therefore, merely be a bystander, a marker of chronic disease or frailty, the result of limited sun exposure. This is, however, a limitation of all of the studies carried out so far and is countered by VINDICATE in which we observed important beneficial left ventricular remodelling with 12 months of vitamin D supplementation [9].

A potential limitation of our study is that although the blood of 25[OH]D vitamin D across centres was assessed using an accepted and validated process, we did not standardise samples across centres. However, decisions on intervention in the form of vitamin D supplementation for CHF patients would be based upon results from local or regional services, abrogating the relevance of this limitation somewhat in real-world practice.

The headline results from the cohort study include data achieved through a robust and recognised multiple imputation process, which aims to reduce the bias introduced by excluding patients with missing 25[OH]D data. The results of the complete case analysis (i.e. without using multiple imputation) are presented in the supplementary materials and describe similar hazard ratios for mortality to those derived from analyses using multiple imputation.

## Conclusion

Even after accounting for potential confounding factors, CHF patients with 25[OH]D sufficiency have a lower risk of all-cause mortality. In conjunction with our recent finding that vitamin D supplementation leads to beneficial cardiac remodelling, these data support the need for a longer term, fully recruited, randomised placebo-controlled study, with ‘hard’ clinical endpoints, of high-dose vitamin D_3_ supplementation in patients with CHF due to left ventricular systolic dysfunction.

### Clinical perspectives

25[OH]D deficiency (< 50 nmol/L) is common in patients with chronic heart failure and persists throughout the year. 25[OH]D deficiency is an independent predictor of higher mortality in patients with CHF on optimal medical and device therapy.

### Implications

Based upon these data and our previous work demonstrating improvements in cardiac function, it is possible that vitamin D_3_ supplementation could improve outcomes in patients with heart failure due to left ventricular systolic dysfunction.

## Electronic supplementary material

Below is the link to the electronic supplementary material.

Supplementary material 1 (DOC 81 KB)

## Abbreviations

CHF: Chronic heart failure
LV: Left ventricular
LVEF: Left ventricular ejection fraction
eGFR: Estimated glomerular filtration rate
NYHA: New York Heart Association
ACEi: Angiotensin-converting enzyme inhibitor
ARB: Angiotensin receptor blocker
ONS: Office of National Statistics
CI: Confidence interval
HR: Hazard ratio
VINDICATE study: Vitamin D treating patients with chronic heart failure
EVITA study: Effect of Vitamin D on all-cause mortality in heart failure study
VIDA study: Vitamin D assessment study
IQR: Interquartile range
PTH: Parathyroid hormone
