# Supplementary material for: Vitamin D deficiency is an independent predictor of mortality in patients with chronic heart failure
Source: Eur J Nutr. 2018 Aug 18;58(6):2535–43. doi: 10.1007/s00394-018-1806-y (PMC6689317; doi:10.1007/s00394-018-1806-y)
Supplement: Supplementary file 1 — Supplementary material 1 (DOC 81 KB) [file 394_2018_1806_MOESM1_ESM.doc]

**Supplementary Table 1:** Imputation model specification for 20 iterations and 30 imputations

| **Variable** | **Type** | **Specification for imputation** |
| --- | --- | --- |
| Nelson-Aalen survival estimate | Continuous (Outcome) | Complete data - included as an auxillary variable for survival time |
| Censoring indicator | Binary (Outcome) | Complete data - included as an auxillary variable |
| Heart failure hospitalisation | Binary (Outcome) | Complete data - included as an auxillary variable |
| Cardiovascular hospitalisation | Binary (Outcome) | Complete data - included as an auxillary variable |
| All non-elective hospitalisation | Binary (Outcome) | Complete data - included as an auxillary variable |
| Gender | Binary | Complete data - included as an auxillary variable |
| Diabetes | Binary | Complete data - included as an auxillary variable |
| COPD | Binary | Complete data - included as an auxillary variable |
| Ischaemic HF Aetiology | Binary | Complete data - included as an auxillary variable |
| Device therapy | Binary | Complete data - included as an auxillary variable |
| Age | Binary | Complete data - included as an auxillary variable |
| Month of recruitment | Continuous | Complete data – included as an auxillary variable |
| Year of recruitment | Continuous | Complete data – included as an auxillary variable |
| NYHA Class | Categorical | Ordinal logistic regression |
| Heart Rate | Continuous | Linear regression |
| QRS interval | Continuous | Log transformed and imputed using linear regression |
| Haemoglobin | Continuous | Linear regression |
| Sodium | Continuous | Predictive mean matching |
| eGFR | Continuous | Linear regression |
| 25[OH]D | Continuous | Log transformed and imputed using linear regression |
| Albumin | Continuous | Linear regression |
| LV End diastolic dimension | Continuous | Linear regression |
| LV Ejection fraction | Continuous | Linear regression |
| Ramipril Dose | Continuous | Predictive mean matching |
| Bisoprolol Dose | Continuous | Predictive mean matching |
| Furosemide Dose | Continuous | Predictive mean matching |

**Supplementary Table 2: Complete case analyses of the association of 25[OH]D with hospitalisation over one year (logistic regression analysis)**

| **Model** | **OR** | **Lower 95% CI** | **Upper 95% CI** | **P value** |
| --- | --- | --- | --- | --- |
| **Heart Failure Hospitalisation** |  |  |  |  |
| Unadjusted | 0.64 | 0.44 | 0.94 | 0.024 |
| Adjusted for patient and clinical demographics^1^ | 0.66 | 0.42 | 1.03 | 0.069 |
| Adjusted for patient and clinical demographics, and comorbidities and aetiology^2^ | 0.68 | 0.43 | 1.06 | 0.086 |
| Adjusted for patient and clinical demographics, comorbidities and aetiology, and treatment^3^ | 0.67 | 0.43 | 1.06 | 0.087 |
|  |  |  |  |  |
| **Cardiovascular Hospitalisation** |  |  |  |  |
| Unadjusted | 0.73 | 0.55 | 0.97 | 0.032 |
| Adjusted for patient and clinical demographics^1^ | 0.78 | 0.57 | 1.08 | 0.132 |
| Adjusted for patient and clinical demographics, and comorbidities and aetiology^2^ | 0.81 | 0.58 | 1.11 | 0.189 |
| Adjusted for patient and clinical demographics, comorbidities and aetiology, and treatment^3^ | 0.80 | 0.58 | 1.11 | 0.180 |
|  |  |  |  |  |
| **All non-elective Hospitalisations** |  |  |  |  |
| Unadjusted | 0.74 | 0.60 | 0.91 | 0.005 |
| Adjusted for patient and clinical demographics^1^ | 0.82 | 0.65 | 1.04 | 0.099 |
| Adjusted for patient and clinical demographics, and comorbidities and aetiology^2^ | 0.83 | 0.65 | 1.05 | 0.117 |
| Adjusted for patient and clinical demographics, comorbidities and aetiology, and treatment^3^ | 0.83 | 0.65 | 1.06 | 0.134 |

^1^Including age, sex, month and year of recruitment, sodium, eGFR, albumin, log transformed QRS interval, NYHA class, LV Ejection Fraction, LV End Diastolic Dimension. ^2^Diabetes, COPD, Ischaemic aetiology. ^3^Ramipril dose, Bisoprolol dose, Furosemide dose, device therapy

**Supplementary Table 3: Complete case analyses of the association of 25[OH]D with all-cause mortality using Cox-proportional hazards**

| **Model** | **HR^**^** | **Lower 95% CI** | **Upper 95% CI** | **P value** |
| --- | --- | --- | --- | --- |
| **All-cause mortality** |  |  |  |  |
| Unadjusted | 0.80 | 0.69 | 0.93 | 0.004 |
| Adjusted for patient and clinical demographics^1^ | 0.84 | 0.71 | 1.00 | 0.050 |
| Adjusted for patient and clinical demographics, and comorbidities and aetiology^2^ | 0.84 | 0.71 | 1.00 | 0.049 |
| Adjusted for patient and clinical demographics, comorbidities and aetiology, and treatment^3^ | 0.87 | 0.73 | 1.04 | 0.122 |

**^**^**HR per 2.72-fold increase in 25[OH]D (due to natural log transformation to achieve normality) ^1^Including age, sex, month and year of recruitment, sodium, eGFR, albumin, log transformed QRS interval, NYHA class, LV Ejection Fraction, LV End Diastolic Dimension. ^2^Diabetes, COPD, Ischaemic aetiology. ^3^Ramipril dose, Bisoprolol dose, Furosemide dose, device therapy.
